# Osteogenic differentiation of skeletal muscle progenitor cells is activated by the DNA damage response

**DOI:** 10.1038/s41598-019-41926-3

**Published:** 2019-04-01

**Authors:** M. Rosina, F. Langone, G. Giuliani, A. Cerquone Perpetuini, A. Reggio, A. Calderone, C. Fuoco, L. Castagnoli, C. Gargioli, G. Cesareni

**Affiliations:** 1 Department of Biology, University of Rome “Tor Vergata”, Rome, Italy; 2 Fondazione Santa Lucia Istituto di Ricovero e Cura a Carattere Scientifico (IRCCS), Rome, Italy

## Abstract

Heterotopic ossification (HO) is a pathological condition characterized by the deposition of mineralized tissue in ectopic locations such as the skeletal muscle. The precise cellular origin and molecular mechanisms underlying HO are still debated. In our study we focus on the differentiation of mesoangioblasts (MABs), a population of multipotent skeletal muscle precursors. High-content screening for small molecules that perturb MAB differentiation decisions identified Idoxuridine (IdU), an antiviral and radiotherapy adjuvant, as a molecule that promotes MAB osteogenic differentiation while inhibiting myogenesis. IdU-dependent osteogenesis does not rely on the canonical BMP-2/SMADs osteogenic pathway. At pro-osteogenic conditions IdU induces a mild DNA Damage Response (DDR) that activates ATM and p38 eventually promoting the phosphorylation of the osteogenesis master regulator RUNX2. By interfering with this pathway IdU-induced osteogenesis is severely impaired. Overall, our study suggests that induction of the DDR promotes osteogenesis in muscle resident MABs thereby offering a new mechanism that may be involved in the ectopic deposition of mineralized tissue in the muscle.

## Introduction

Heterotopic ossification (HO) is a pathological condition causing the formation of ectopic bone in soft tissues. The etiology of this phenomenon is imputable to genetic or physio-pathological alterations^1,2^ including different types of injuries. Traumatic or sport-derived injuries, neuropathic disorders and severe burns can cause^3–5^ a localized inflammatory response, mediated by myeloid cells and lymphocytes, which in turn produces high levels of osteogenic cytokines, such as TGFβ2 and BMP4 promoting ectopic bone formation^6–8^. Moreover mutations in the ACVR1 gene, encoding for BMP type I receptor, are frequent in patients affected by *fibrodysplasia ossificans progressiva* (FOP), a rare and catastrophic disease leading to progressive ossification of many soft tissues of the body^9^.

Osteogenic differentiation is physiologically mediated by the bone morphogenetic protein (BMP) signaling pathway. BMPs are members of the TGFβ cytokine superfamily activating two independent signaling cascades, by binding to the serine/threonine kinase receptors BMPRII and BMPRI^10^. In a first path the phosphorylation of receptor-activated SMAD1-5-9 (R-SMADs) activates the Runt-related transcription factor 2 (RUNX2)^11^, which is the master regulator of osteogenic differentiation^12^. In a parallel path, RUNX2 is activated by the p38 MAPK^13^. RUNX2 in turn activates the expression of the transcription factor SP7, responsible for the up-regulation of late osteogenic markers^14^.

The cell type promoting heterotopic ossification in the skeletal muscle tissue is still debated^15,16^. Adult mesoangioblasts (MABs) are multipotent perivascular stem/progenitor cells of mesenchymal origin, that have the potential to differentiate into different mesodermal cell types, including skeletal and smooth muscle, osteoblasts and adipocytes^17^.

Here we investigate the mechanisms modulating MAB osteogenesis by performing a high content screening to identify small molecules triggering osteogenic differentiation.

We report that 5-iodo-2′-deoxyuridine (IdU) is a potent inducer of MAB osteogenesis. The mechanism is SMAD1-5-9 independent and p38-dependent. Finally, we show that IdU incorporation into the DNA triggers the DNA damage response (DDR) and that the inhibition of the DNA damage responsive kinase ATM can hinder osteogenic differentiation. We propose that activation of the DDR in mesenchymal progenitor cells may cause heterotopic ossification *in vivo*.

## Results

### Idoxuridine induces osteogenesis and limits the myogenic differentiation

**Figure 1:**
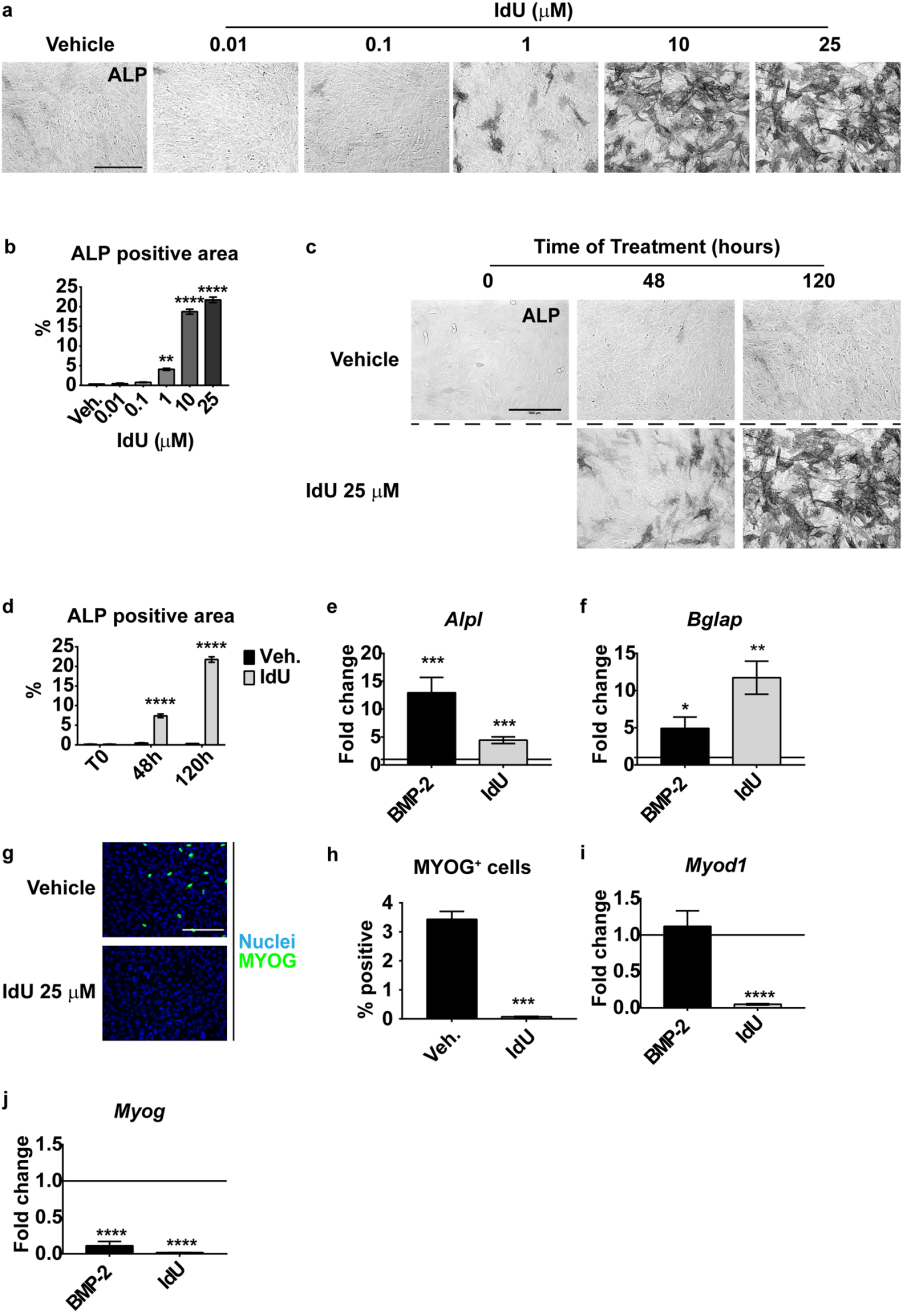
IdU treatment promotes osteogenic differentiation and inhibits myogenesis. (**a**) Alkaline phosphatase (ALP) staining of IdU-treated MABs. MABs were treated with increasing concentrations of IdU and incubated for 120 hours in growth medium. Scale bar 200 μm. (**b** and **d**) Quantitation of ALP positive area in the experiment in panel (a and c) respectively. Data are presented as the percentage of ALP positive area over the total area ± SEM. Statistical significance was assessed by a One-Way Anova (panel b) or Two-Way Anova (panel d). **p < 0.01; ****p < 0.0001; n = 3. (**c**) Differentiation time-course. MABs were treated with 25 μM IdU in growth medium and samples were harvested at 48 hours and 120 hours. Scale bar 200 μm. (**e** and **f)** Real time PCR analysis of *Alpl* and *Bglap* genes upon IdU and BMP-2 treatment at 120 hours. Fold change calculation was performed using the 2^−ΔΔCt^ method relative to vehicle treated sample (horizontal line = 1). Data are presented as means ± SEM. Statistical analysis was performed by a Student’s t-test. *p < 0.05; **p < 0.01; ***p < 0.001; n = 6. (**g**) Myogenic differentiation of MABs treated with 25 μM IdU. MABs were cultivated for 120 hours in growth medium to allow spontaneous myogenic differentiation. Scale bar 200 μm. (**h)** Quantitation of MYOG positive nuclei in panel (g). Data are presented as percentage of MYOG positive nuclei over the total number of nuclei per field ± SEM. Statistical significance was assessed by a Student’s t-test. ***p < 0.001; n = 3. (**i** and **j**) Real time PCR analysis of *Myod1* and *Myog* genes upon IdU and BMP-2 treatment at 120 hours. Fold change calculation was performed using the 2^−ΔΔCt^ method relative to vehicle treated sample (horizontal line = 1). Data are presented as means ± SEM. Statistical analysis was performed by a Student’s t-test. ****p < 0.0001; n = 3 (*Myod1*); n = 4 (*Myog*).

Mesoangioblasts (MABs) are multipotent myogenic progenitor cells that spontaneously undergo muscle differentiation, forming myotubes. If exposed to the Bone Morphogenic Protein 2 (BMP-2) they switch their differentiation program forming osteoblasts^18^. To identify cell perturbations that would promote MAB osteogenesis, we have developed a robust high-throughput, high-content screening by monitoring the activity of alkaline phosphatase (ALP) and myogenin expression. We performed the primary screening in the presence of 0.3 μg/ml of BMP-2. This stimulus is not sufficient to promote the formation of alkaline phosphatase positive MABs but might contribute to reveal molecules that act synergistically with BMP-2. Out of 560 compounds of the Prestwick library of FDA approved drugs, 13 small molecules (2,3% of total molecules screened) displayed some ability to induce MAB differentiation into osteoblasts when administrated in the presence of low BMP-2 concentrations. The myogenin staining also allowed to identify compound that promote (8 molecules) or inhibit (16 molecules) MAB myogenesis. The primary screening hits were retested, in the conditions of the primary screening. The compounds whose activity was confirmed in the secondary screening are listed in Supplementary Tables ST1–ST3. We identified a single drug, Idoxuridine (IdU), which significantly induces ALP activity, in a dose-dependent manner with an IC50 of approximately 5 μM (Fig. 1a,b) even in the absence of BMP-2. ALP accumulation is apparent after 48 hours of IdU exposure and reaches a maximum at 120 hours (Fig. 1c,d). IdU induces the expression of both *Alpl* (Alkaline phosphatase) and *Bglap* (Bone gamma-carboxyglutamate protein/osteocalcin) mRNAs, late markers of osteogenic differentiation (Fig. 1e,f). IdU not only promotes osteogenesis but also interferes with myogenesis, as observed by staining with anti-myogenin fluorescent antibody and by real time PCR analysis for *Myod1* and *Myog* (Fig. 1g–j). Notably, IdU treatment does not affect cell proliferation at the tested concentrations (Fig. S1a), suggesting that the inhibition of myogenic differentiation is cell confluence-independent. Moreover, both IdU and BMP-2 treatments cause opposite modulation of osteogenic and myogenic genes.

### IdU treatment induces the expression of the osteogenic transcription factor Runx2

We next looked at the expression of *Runx2* and *Sp7*, two master regulators of transcription of osteogenic genes. Since *Runx2* expression and activity are controlled both at transcriptional and post-translational levels^19^, we assessed the modulation of both mRNA and protein levels after IdU treatment.

**Figure 2:**
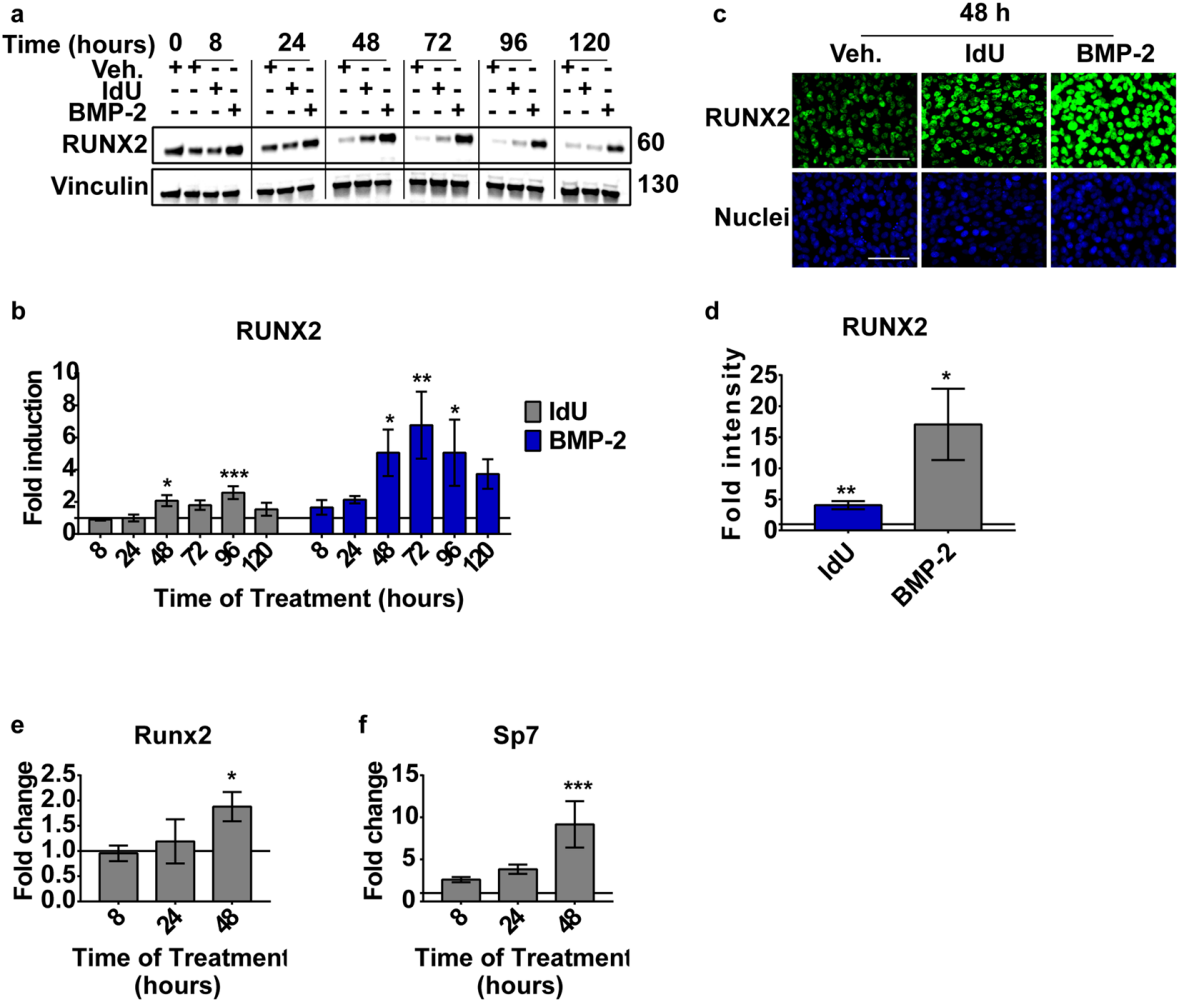
IdU treatment induces the expression of the osteogenic transcription factors. (**a**) Representative western blot of MABs treated with vehicle, 25 μM IdU or 1 μg/ml BMP-2. The complete gel is reported in Figure S5 (**b)** Densitometric quantitation of western blots as in panel (a). Vinculin was used as normalizer and data are presented as fold induction ± SEM relative to vehicle treated sample at each time point (horizontal line = 1). Statistical significance was assessed by a One-Way Anova test. *p < 0.05; **p < 0.01; ***p < 0.001; n = 3. (**c**) Representative anti-RUNX2 immunofluorescence of MABs treated for 48 hours with vehicle, 25 μM IdU or 1 μg/ml BMP-2. Scale bar is 100 μm. (**d)** Fluorescence intensity quantitation of images in panel (c). Fold intensity is relative to vehicle-treated sample (horizontal line = 1). Data are presented as mean ± SEM. Statistical analysis was performed by a Student’s t-test. *p < 0.05; **p < 0.01; n = 3. (**e**) Real time PCR analysis of *Runx2* and **f)** *Sp7* in MABs treated with vehicle or 25 μM IdU. Fold change calculation was performed using the 2^−ΔΔCt^ method relative to vehicle treated sample (horizontal line = 1). Data are presented as means ± SEM. Statistical analysis was performed by a Two-Way Anova test. *p < 0.05; ***p < 0.001; n = 3.

In IdU-treated cells the RUNX2 transcription factor is overexpressed starting at 48 hours, compared to controls. However, its expression levels, as assessed both by western blot and by immunofluorescence staining, remain lower in comparison to that of cells induced to differentiate with BMP-2 (Fig. 2a–d). To further investigate the early phases of this process, we performed real time PCR analysis confirming a similar kinetic of accumulation of both *Runx2* and *Sp7* mRNA levels (Fig. 2e,f). In addition we found that IdU treatment does not induce the expression of *Sox9* (Fig. S1b), the master regulator of chondrogenic differentiation^20^.

We conclude that IdU treatment promotes osteogenic differentiation by inducing the expression of the master transcription factors that regulate osteogenesis. We next asked whether the mechanisms inducing the expression of Runx2 were similar in BMP-2 and IdU-treated cells.

### IdU promotes osteogenesis by a BMP-independent/p38-dependent pathway

**Figure 3:**
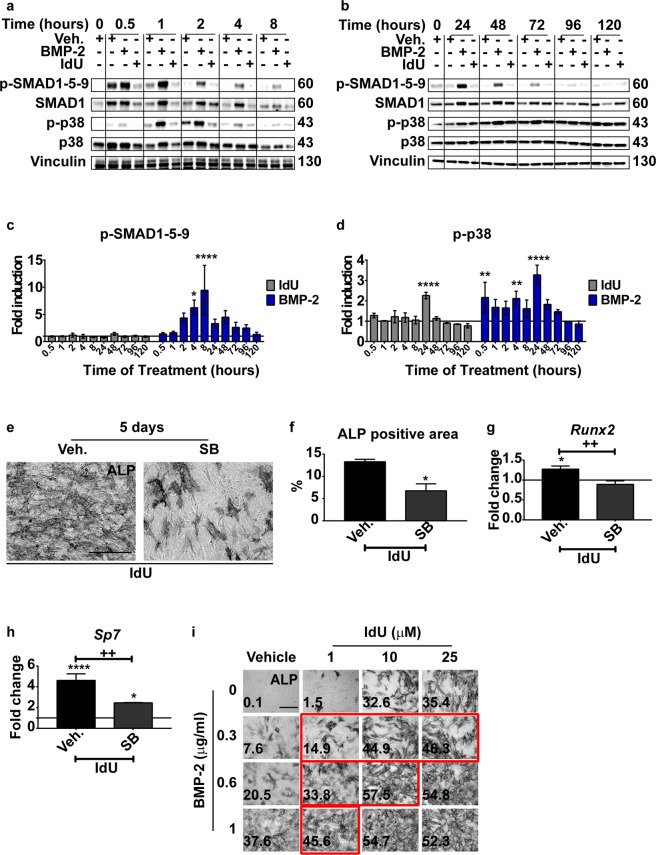
Analysis of SMAD1-5-9 and p38 activation upon IdU treatment. Representative western blot at (**a)** early and (**b**) late time points of p-SMAD1-5-9 and p-p38. The complete gels are available at Figures S6 and S7 for panel (a) and (b) respectively. (**c,d)** Densitometric quantitation of bands in panel (a) and (b). Data are represented as fold induction relative to vehicle-treated sample (horizontal line = 1). Statistical significance was assessed by a Two-Way Anova test. **p < 0.01; ***p < 0.001; ****p < 0.0001; n = 3. (**e)** Representative image of ALP staining of MABs pre-treated with vehicle or 10 μM SB 203580 for 3 hours in growth medium and then treated with 25 μM IdU in combination with 10 μM SB 203580 for 5 days in growth medium. Scale bar 200 μm. (**f)** ALP positive area quantitation of images in panel (e). Data are presented as percentage of ALP positive area over the total area of the image filed ± SEM. Statistical significance was assessed by a Student’s t-test. *p < 0.05; n = 3. (**g,h)** Bar plot of real time PCR analysis of *Runx2* and *Sp7* of MABs pre-treated with vehicle or 10 μM SB 203580 for 3 hours in growth medium and then treated with 25 μM IdU in combination with 10 μM SB 203580 for 48 hours in growth medium. Data are presented as fold change versus untreated sample (horizontal line = 1) ± SEM. Statistical analysis was performed by a Two-Way Anova. “Asterisk” symbol (*) represents the significance versus CTRL sample. *p < 0.05; ****p < 0.0001. “Plus” symbol (+) represents the significance between Vehicle and SB pre-treated sample. ^++^p < 0.01. n = 3. (**i)** ALP staining of MABs treated with IdU in combination with BMP-2. MABs were treated and cultivated for 120 hours in growth medium. Osteogenic differentiation was assessed by ALP staining. ALP positive area percentage is reported at the bottom-left side of each micrograph. Red-squared images show an additive effect between BMP-2 and IdU. The complete quantitation of ALP staining is shown in Figure S2.

To assess the molecular mechanism underlying IdU-induced osteogenesis we first looked at the canonical osteogenic pathway. Bone morphogenetic protein 2 (BMP-2) is one of the physiological inducers of osteogenesis in osteoblast precursors. BMP-2 induces osteogenic differentiation by the SMAD and p38-dependent pathways^19^. To evaluate if these pathways are also activated after IdU treatment we monitored the phosphorylation of both SMAD1-5-9 and p38 at early and late time points throughout the differentiation process. IdU does not induce any phosphorylation of SMAD1-5-9 at any time point indicating that IdU promotes osteogenesis by a SMAD-independent pathway (Fig. 3a,b,c). On the other hand, an acute and transient activation of p38 is observed at 24 hours (Fig. 3b,d). Conversely, IdU treatment does not induce the activation of the ERK/MAPK-dependent signaling cascade (Fig. S2a,b). To confirm that activation of p38 is involved in IdU induction of osteogenesis we added the p38 kinase inhibitor SB 203580 to IdU-treated MABs. A significant reduction of ALP expression is observed in presence of the kinase inhibitor (Fig. 3e,f). The decrease of ALP expression after p38 inhibition is paralleled by a downregulation of *Runx2* and *Sp7* gene transcripts (Fig. 3g,h).

We next asked whether the mechanisms inducing the expression of p38 were similar in BMP-2 and IdU treated cells. Compounds that induce the same phenotypic transition via alternative mechanisms are more likely to show synergy^21^. We asked whether IdU synergizes with BMP-2 in the induction of MAB osteogenesis. MABs were treated in combination with BMP-2 and IdU at different concentrations. Co-treatment with both substances achieved a better than additive effect when compared to the differentiation obtained with the same concentrations of the single substances, as revealed by the quantitation of ALP expression (Fig. 3i, S2c). Two-Way ANOVA statistics confirmed the synergic effect between BMP-2 and IdU showing a strong “Interaction” effect between the two groups (Supplementary Table ST4).

We conclude that the late and transient induction of p38 is essential for IdU-mediated osteogenesis, while IdU treatment does not seem to require the activation of the SMAD signaling cascade. The synergic effect with BMP-2 prompted us to hypothesize that the upstream signals leading to p38 activation after IdU treatment are dependent on a non-canonical signaling pathway.

### Idoxuridine incorporates into DNA and affects the cell proliferation rate by inducing a delay at the G2-M transition

**Figure 4:**
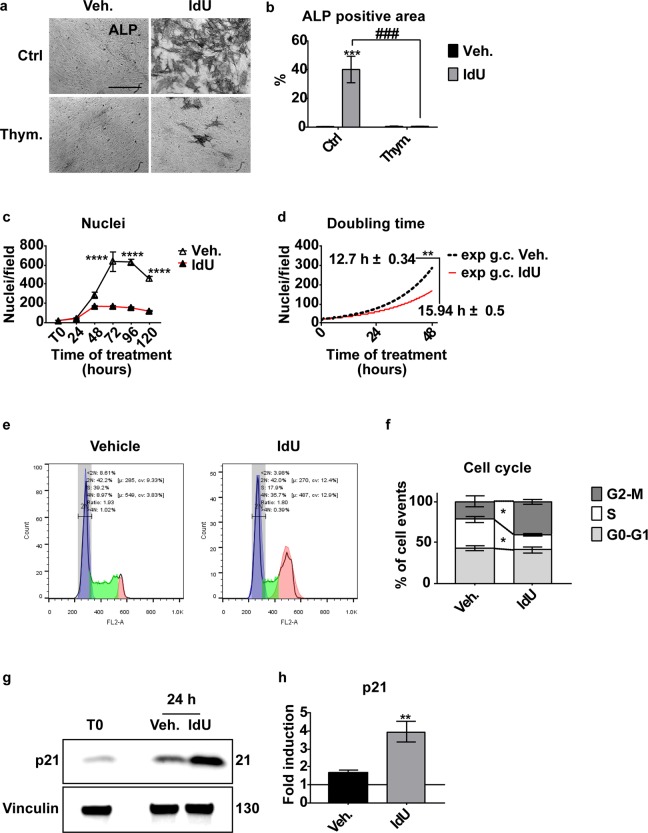
MABs proliferation and cell cycle analysis upon IdU treatment at low seeding density. **(a)** Representative image of the thymidine-competition assay. MABs were treated with 25 μM IdU, 25 μM thymidine and in combination, harvested after 5 days and stained for alkaline phosphatase activity. Scale bar 200 μm. (**b)** ALP positive area quantitation of images in panel (a). Data are presented as percentage of ALP positive area over the total area of the image filed ± SEM. Statistical significance was assessed by a Two-Way ANOVA test. ***p < 0.001; ^##^p < 0.01; n = 3. (**c)** Quantitation of MABs nuclei stained with Hoechst 33342 in control cultures and in cultures treated with 25 μM IdU. Data are presented as mean of nuclei per field ± SEM. Statistical significance was assessed by a Two-Way Anova test taking Vehicle treated sample as reference. ****p < 0.0001; n = 3. (**d**) Exponential growth analysis of data in panel (c). The doubling time of Vehicle and IdU treated MABs was analyzed with Non-linear regression and Exponential growth equation tool of Graph Pad Prism 6. Doubling time is presented as mean ± SEM. The statistical significance between the two doubling time data sets was assessed by a Student’s t-test. **p < 0.01. n = 3 (**e**) Analysis of single cell DNA content as measured by cytofluorimetry of propidium iodide stained cells. 10,000 cell events were analyzed for each sample. Data are presented as counts over FL2-A fluorescence. Diploid nuclei in blue. S phase in green. Tetraploid nuclei in red. (**f)** Quantitation of cell cycle analysis. Data are presented as percentage of events ± SEM. Statistical significance was assessed by a Two-Way Anova test. *p < 0.05; n = 3. (**g)** Western blot for p21 of 25 μM IdU treated MABs at time zero (T0) and 24 hours after treatment. Vinculin was taken as loading control. The complete gel is reported in Figure S8. (**h)** Densitometric quantitation of p21 in panel (g). Values were normalized over vinculin and the fold induction was calculated over the time zero (T0 = 1) sample. Data are represented as mean ± SEM. Statistical significance was assessed by a Student’s t-test. **p < 0.01. n = 3.

The MAPK p38 is involved in many cellular processes such as control of staminality and differentiation^22,23^. Moreover, p38 is a key player in DNA damage stress response and cell cycle control^24^. Since IdU incorporation into DNA interferes with DNA synthesis^25,26^, we wondered whether it may cause perturbation of the cell cycle. We first asked whether IdU affects MAB differentiation by being incorporated into DNA. To this end, we performed a competition experiment exposing cells to 25 μM IdU and 25 μM thymidine, its physiological analogue. In the co-treatment condition, ALP activity is significantly reduced in comparison with samples treated with IdU only (Fig. 4a,b). Thus, by limiting incorporation of IdU into the DNA via addition of equimolar concentration of thymidine, osteoblast differentiation is severely hampered suggesting that IdU affect differentiation by incorporating into DNA.

Taken together, these results are consistent with the hypothesis that p38 activation, as a consequence of IdU incorporation into MAB DNA, negatively impact cell proliferation, while promoting osteogenic differentiation. To lend further support to this hypothesis we investigated the perturbations of MAB cell cycle induced upon IdU treatment.

When seeded at a density of 3.1 × 10^3^ cells/cm^2^, IdU-treated cells arrest their growth after 48 hours, while control cells grow exponentially for 72 hours (Fig. 4c, S3a). In the exponential growth phase, the proliferation rate of IdU-treated MABs is lower than in the control (Fig. 4d).

To further analyze the cell cycle dynamics, we looked at the distribution of the cell populations in the different phases of the cell cycle. After 24 hours of IdU treatment we observe a reduction in the fraction of cells in the S phase and an increment in the fraction of cells arrested at the end of the G2 phase (Fig. 4e,f). In accordance, p21, a marker of the cell cycle arrest, accumulates at 24 hours after treatment (Fig. 4g,h).

### IdU-induced osteogenesis is dependent on the DNA damage response

**Figure 5:**
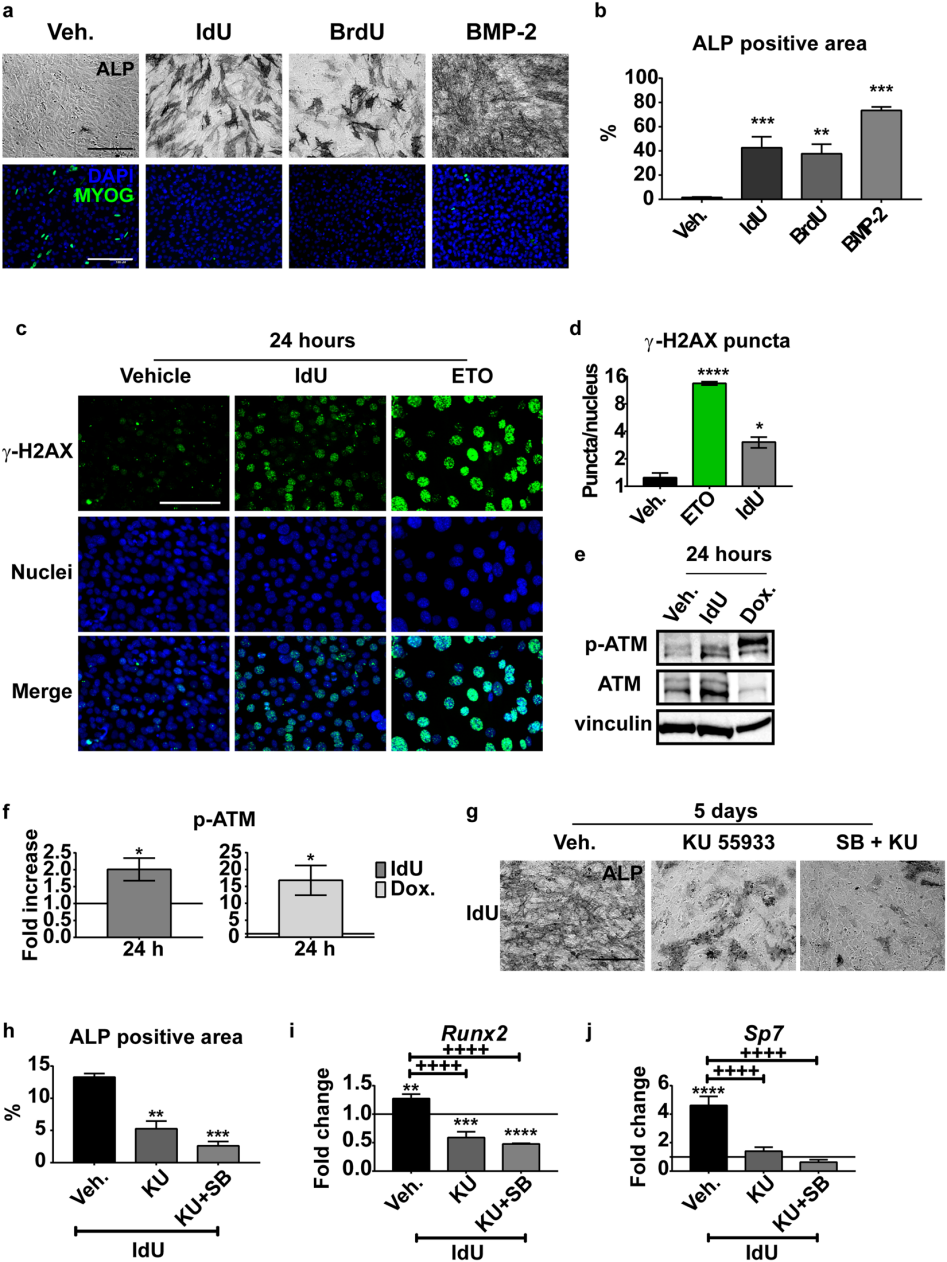
IdU induces in MABs a DNA Damage Response upon IdU treatment. (**a**) Representative images of alkaline phosphatase staining and anti-MYOG immunofluorescence of IdU, BrdU and BMP-2 treated MABs. Scale bar 200 μm. (**b**) ALP positive area quantitation of images in panel (a). Data are presented as percentage of ALP positive area over the total area of the image filed ± SEM. Statistical significance was assessed by a One-Way ANOVA test. **p < 0.01 ***p < 0.001; n = 3. (**c)** immunofluorescence analysis of γ-H2AX. MABs were treated with 25 μM IdU or 0.5 μM Etoposide (ETO) as positive control and fixed after 24 hours. Scale bar is 100 μm. (**d**) Bar plot of the quantitation of γ-H2AX nuclear puncta of images in panel (c). Data are represented as the number of nuclear puncta per nucleus ± SEM considering all the nuclei in the image field. Statistical analysis was performed with One-Way Anova. *p < 0.05; ****p < 0.0001. n = 3. (**e**) Representative western blot of p-ATM and ATM upon IdU treatment. MABs were treated with 25 μM IdU or 0.5 μM Doxorubicin (Dox.) as positive control and harvested after 24 hours. Vinculin was used as loading control. The complete gel is reported in Figure S9. (**f)** Bar plot of densitometric quantitation of bands in panel (e). Data are represented as mean ± SEM of fold increase relative to vehicle (Veh.) treated sample. Statistical analysis was performed through the Student’s t-test. *p < 0.05. n = 3. (**g)** ALP staining of MABs treated with 25 μM IdU after pre-treatment with 10 μM KU 55933 ATM inhibitor alone or in combination with 10 μM SB 203580 p38 inhibitor. Scale bar is 200 μM. (**h**) Bar plot of ALP positive area quantitation of images in panel (g). Data are represented as mean ± SEM of the percentage of ALP positive area of the total area of the image. Statistical analysis was performed through One-Way Anova. ** p < 0.01. n = 3. (**i,j)** Bar plot of RT-qPCR analysis of *Runx2* and *Sp7* of MABs pre-treated with vehicle or 10 μM KU 55933 ATM inhibitor alone or in combination with 10 μM SB 203580 p38 inhibitor for 3 hours in growth medium and then treated with 25 μM IdU for 48 hours in growth medium. Data are represented as fold change versus untreated sample (CTRL = 1) ± SEM. Statistical analysis was performed through Two-Way Anova. “Asterisk” symbol (*) represents the significance versus CTRL sample. **p < 0.01; ***p < 0.001; ****p < 0.0001. “Plus” symbol (+) represents the significance between DMSO and SB pre-treated sample. ^++++^p < 0.0001. n = 3.

Given that the MAB osteogenic potential is positively modulated by IdU incorporation into the DNA, we checked whether a different non-natural nucleotide analog, 5-bromo-2′-deoxyuridine (BrdU) would display a similar activity. As shown in Fig. 5a,b, BrdU was also able to induce MAB osteogenesis albeit to a lower extent in comparison to IdU (Fig. 5a,b). Similarly to IdU, BrdU also inhibited myogenesis (Fig. 5a, S4a). In addition, treating MABs with 25 μM uridine or other DNA-interacting molecules (as 0.5 μM etoposide and cisplatin) does not cause osteogenic differentiation (Fig. S4b). This suggests that the incorporation of nucleotide analogs into the DNA, possibly by disturbing the double helix structure, could trigger a signaling cascade that modulates MAB differentiation decisions. Consistently, immunofluorescence staining of phosphorylated H2AX (γ-H2AX), 24 hours after treatment with IdU, shows an increase of γ-H2AX nuclear puncta (Fig. 5c,d), which is, however, less prominent than the one observed after treatment with the DNA damaging agent etoposide (ETO). Western blot analysis confirmed the activation of the upstream ATM kinase, which requires phosphorylation of γ-H2AX after IdU treatment. (Fig. 5e,f).

Interestingly, the ATM kinase inhibitor KU 55933, negatively modulates the induction of ALP as well as the transcription of *Runx2* and *Sp7*. This inhibition is further reinforced by the addition of the p38 kinase inhibitor SB 203580 (Fig. 5g–j). Taken together, these results are consistent with a model whereby the osteogenic differentiation of MABs upon IdU treatment is dependent on the activation of the DDR-related ATM/p38 axis.

## Discussion

Under a variety of stimuli, including inflammation and traumatic injury, skeletal muscle residing mesenchymal stem cells differentiate into osteoblast precursors^1^. This event, dubbed as heterotopic ossification (HO), causes the formation of bone foci inside the muscle parenchyma thereby affecting its function. Heterotopic ossification is a debilitating condition whose etiology is not fully understood. Although the identity of progenitor cells that promotes the formation of heterotopic bones and cartilage is still debated, cells expressing vascular endothelial markers have been proposed as candidate originators of this aberrant process^15^.

Adult mesoangioblasts (MABs) are muscle resident multipotent mesenchymal progenitors that are physically associated to blood vessels^27^. Once isolated from the muscle tissue, they have the potential to differentiate into skeletal and smooth muscle or osteoblasts^18^. With the aim of characterizing perturbations and pathways that control the differentiation choice of muscle precursor cells we developed an assay for osteoblastic differentiation suitable for high-content screening. This approach allowed us to identify Idoxuridine (IdU) as an inducer of MAB osteogenic differentiation as inferred by the expression of the early osteogenic marker alkaline phosphatase. IdU treated MABs, however, also express additional bone proteins as for instance osteocalcin, a late osteogenic marker involved in the production of the extracellular mineralized matrix^28^. *Bglap*, the osteocalcin encoding gene is transcribed as early as five days after treatment in our conditions. This is comparable with the kinetic reported in other *in vitro* differentiation models where the mRNA of *Bglap* is already detectable at day 4 and reaches a maximum after 21 days from induction of osteogenesis^29^.

Osteogenesis is physiologically controlled by the BMP-SMAD signaling pathway. In such path, the BMP binds to its serine/threonine-kinase receptor and triggers a phosphorylation cascade that activates the transcriptional activity of the osteogenic transcription factor RUNX2. In parallel, the same receptor induces a MAP kinase cascade through the activity of the MAP kinase-kinase 3-6 (MKK 3–6) and p38^10,11^. The activation of p38 is important for the induction of the osteogenic program, since RUNX2 transcriptional activity is promoted by phosphorylation by this MAPK on multiple residues^10,11,13,30^. BMP-2 and IdU, when co-administered, show a more than additive effect in the induction of osteogenic differentiation (Fig. 3i, S2c, Supplementary table ST4) suggesting that the two stimuli act via different mechanisms and pathways.

Consistently, we show that IdU induces the expression of both *Runx2* and *Sp7* (Fig. 2) independently from the activation of the BMP/SMAD signaling pathway (Fig. 3a–c), while an acute but transient activation of p38 is observed in a limited time window (Fig. 3b,d). The osteogenic phenotype and the expression of the related transcription factors after IdU treatment, are strictly dependent on the activation of p38, as shown by their sensitivities to p38 inhibitors (Fig. 3e–h).

Additional members of the MAPKs protein family, such as ERK 1–2 and JNK have been implicated in osteoblast differentiation and bone homeostasis^31^. The deletion of ERK 1–2 causes defective bone mineralization^32^. ERK activates the transcriptional activity of RUNX2 by phosphorylating S301 and S319^33^. IdU on the other hand does not activate the ERK pathway (Fig. S2a,b). The activity of the JNK MAPK was also linked to the late stages of osteoblasts differentiation, according to Yamashita and Guicheux showing a decrease in ALP and osteocalcin expression during osteoblast differentiation, upon JNK inhibition^34,35^.

IdU is a nucleotide analogue that is prescribed as an antiviral agent given its ability to interfere with the DNA synthesis of *Herpes simplex* virus^26,36^. Moreover, the incorporation of IdU in eukaryotic cells sensitizes cancer cells to radiotherapy treatment, by inducing DNA double strand breaks (DSBs) on highly proliferative cells^25,37,38^.

Our results support a model whereby IdU induces a cell cycle delay in the G2-M transition phase (Fig. 4). Cell cycle modulation is a critical step for osteoblast differentiation. Lau and colleagues showed that low doses of X-irradiation induce G2-arrest in the pre-osteoblast cell line OCT-1 through the activation of p21 and the subsequent induction of *Runx2* expression^39^. This cell cycle perturbation could be imputed to the activation of the p38 axis. Upon DNA damage ATM activates p38 through the coordinated action of the Thousand And One amino acid protein 1–3 (TAO1–3) and the MAP Kinase-Kinase 3–6 (MKK3-6). The induction of this signaling cascade results in the inhibition of the CDC25c phosphatase, resulting in the cell cycle arrest at the G2-M transition^40,41^. The observation that, in analogy with IdU, also the halogenated nucleotide BrdU promotes osteogenic differentiation (Fig. 5a,b) prompted us to investigate whether incorporation of nucleotide analogs would induce DNA damage causing a DNA Damage Response (DDR).

The activation of the ATM kinase is the first response of the DDR to DSBs and it is controlled by an autophosphorylation event^42^ that triggers its kinase activity on downstream targets, as the histone isoform H2AX^43^ (Fig. 5b–f). γ-H2AX in turn plays an important role in the recruitment and assembly of the DNA repair molecular machinery^44,45^. The ATM kinase has already been implicated in osteogenic differentiation. Rasheed *et al*. speculated that ATM could positively modulate the BMP pathway, since ATM ^−/−^ mice display an osteoporotic phenotype due to the detrimental effect on the expression of *Sp7* transcription factor^46^. By contrast, here we show that IdU treatment activates ATM and induces osteogenesis by a BMP/SMAD-independent pathway. It has been reported that the osteogenic differentiation could also be controlled through RUNX2-independent pathways. Lee and Ulsamer characterized a Dlx5-dependent/Runx2-independent mechanism directly acting on the expression of Sp7^47,48^. Dlx5 is phosphorylated by p38 on residues S34 and S217 to increase its transactivation activity. In our system IdU treatment does not promote Dlx5 expression (data not shown).

In our study, we show that IdU-induced osteogenesis requires both ATM and p38 as pharmacological inhibition of either kinase activity reduces the transcriptional activation of both *Runx2* and *Sp7* and, consequently, osteogenic differentiation (Figs 3e–h, 5g–j).

Overall, our data point to DDR as a potential modulator of the differentiation fate of skeletal muscle resident mesenchymal stem cells. In particular we show that in MABs the DDR can influence the differentiation “choice” between the myogenic and the osteogenic fate.

The observation that p53 ^−/−^ and c-Abl ^−/−^ mice are characterized by an osteosclerotic and an osteoporotic phenotype, respectively, points to the involvement of these two direct targets of ATM and of the DNA repair pathway^49–51^ in osteogenic differentiation and bone development.

In direct contrast with the role of ATM in promoting osteoblast differentiation is the recent evidence of the ATM negative impact in the activation of the checkpoint kinase Chk2^52^. Chk2 deletion can partially rescue the osteogenic defects in mice lacking the parathyroid hormone-related peptide (PTHrP). Overall, these contrasting evidences highlight a Janus-like effect of ATM, stressing the role of context on its positive or negative effect on osteogenesis.

DNA damage occurs both in physiological and pathological conditions, such as aging^53,54^ and acute or chronic inflammation^55,56^, conditions that also promote ectopic ossification. *Myositis ossificans*, as well as other myo-pathologies such as Duchenne Muscular Dystrophy (DMD), are characterized by an inflammatory response^1,57–59^. Recent evidences suggest a link between HO and the latest complications of DMD^60,61^. Here, we suggest that the inflammation-induced DDR could be one of the key factors in the development of ectopic ossification opening the possibility to develop new therapeutic strategies to counteract this pathological differentiation phenomenon.

## Methods

### Cell culture

Mesoangioblasts (MABs) were kindly provided by Giulio Cossu’s laboratory^62–64^. MABs were seeded on Falcon dishes (Corning) and cultivated in controlled conditions at 37 °C in 5% CO_2_ atmosphere. Growth Medium (GM) consists of Dulbecco’s Modified Eagle Medium (DMEM) GlutaMAX supplement (Thermo Fisher Scientific – #61965) supplemented with 10% v/v heat inactivated Fetal Bovine Serum (FBS) (Euroclone, #ECS0180L), 1 mM sodium pyruvate (Sigma Aldrich, #S8636), 10 mM Hepes (Sigma Aldrich, #H0887) and 100 U/ml penicillin/100 μg/ml streptomycin (Thermo Fisher Scientific, #15140122). Differentiation medium (DM) for the screening procedure consists of the same base medium, 5% v/v FBS and same supplements. For western blot and immunofluorescence assays cells were seeded at the density of 3.5 × 10^4^ cells/cm^2^. For cell growth assay, cytofluorimetry and anti-p21 western blot cells were seeded at the density of 3.1 × 10^3^ cells/cm^2^.

### Screening procedure

Mesoangioblasts (MABs) were plated in 96-well plates at a density of 10,000 cells per well in DM with 0.3 μg/ml bone morphogenetic protein-2 (BMP-2) (R&D Systems, #355-BM). Chemicals were added in the differentiation medium at 1 μM, 10 μM and 25 μM final concentration. Every 96-well plate in the screen included 9 positive controls (3 wells with 0.3 μg/ml of BMP-2, 3 wells with 0.6 μg/ml of BMP-2, 3 wells with 1 μg/ml of BMP-2) and 10 negative controls, where BMP-2 was not added to the medium. The cells were maintained in compounds until day 5. Samples were subsequently stained for alkaline phosphatase activity and with anti-myogenin antibody as described below.

### Treatments and differentiation

All treatments and differentiation were performed in GM. Idoxuridine (IdU) and bromo-deoxyuridine (BrdU) were purchased from Sigma-Aldrich (#I7125, #B5002) and dissolved in DMSO at final concentration of 25 mM. Thymidine (Sigma-Aldrich, #T5018) and uridine (Sigma-Aldrich, #U3750) were dissolved in PBS at final concentration of 25 mM. Recombinant BMP-2 were purchased from Pepro-Tech (#120–02) and prepared according to manufacturer’s instructions to final concentration of 200 μg/ml. Experimental concentrations are indicated in *Figure legends* and *Results*.

Spontaneous myogenic differentiation was obtained by incubating the cells for additional 5 days after reaching confluence.

The induction of DNA damage was performed by treating cells with 0.5 μM Etoposide (Teva Pharmaceuticals) or 0.5 μM Doxorubicin (Sigma Aldrich, #D1515) for 24 hours. Cisplatin was purchased from Hospira (Lot #Y091881AB; Brussel, Belgium) and used at final concentration of 0.5 μM.

ATM and p38 kinase inhibitors, KU 55933 (Tocris Bioscience, #3544) or SB 203580 (Sigma Aldrich, #S8307) respectively, were used at 10 μM in pre-treatment for 3 hours followed by co-treatment with IdU for the indicated time.

### Alkaline phosphatase activity staining

Cells were fixed with paraformaldehyde solution 4% v/v in PBS (Santa Cruz Biotechnology, #sc-281692) for 10 minutes at room temperature (RT) and then washed 3 times with PBS and once with dd-H_2_O.

Specimens were incubated with NBT/BCIP solution (Sigma Aldrich, #11681451001) diluted 1:50 v/v in alkaline buffer (0.1 M Tris-HCl, pH 9.5, 0.1 M NaCl, 0.05 M MgCl_2_) for 10 minutes at RT in the dark and then washed 3 times with PBS.

Specimens were maintained at 4 °C in PBS supplemented with 0.02% w/v sodium azide.

### Immunofluorescence

Cells were fixed with paraformaldehyde solution 4% v/v in PBS for 10 minutes at room temperature (RT) and then washed 3 times with PBS. Permeabilization was performed with 0.5% v/v Triton X-100 in PBS for 5 minutes at RT and washed 3 times with 0.1% v/v Triton X-100 in PBS (washing solution – WS).

For the anti-RUNX2 immunolabeling, permeabilization was performed by incubating cells with methanol for 10 minutes at −20 °C and washed once with dd-H_2_O and three times with PBS before blocking. Blocking was performed with 10% v/v FBS in WS (blocking solution – BS) for 1 hour at RT.

Immunolabeling was carried out in BS for 1 hour at RT with the following antibodies and dilutions: mouse anti-myogenin (F5D) (Thermo Scientific, #14-5643-80, 1:300 v/v), rabbit anti-RUNX2 (D1L7F) (Cell Signaling Technology, #12556, 1:1000 v/v), mouse anti-phospho-Histone H2A.X (Ser139) Antibody (JBW301) (Merck Millipore, #05–636, 1:300 v/v).

After incubation with the primary antibodies the specimens were washed 3 times with WS. Secondary antibodies were incubated for 30 minutes at RT in the dark using the following products and dilutions in BS: Goat anti-Mouse IgG (H + L) Cross-Adsorbed Secondary Antibody, Alexa Fluor 488 (Thermo Fisher Scientific, #A-11001, 1:300 v/v), Goat anti-Rabbit IgG (H + L) Cross-Adsorbed Secondary Antibody, Alexa Fluor 488 (Thermo Fisher Scientific, #A-11008, 1:300 v/v).

After the incubation with the secondary antibodies the specimens were washed 3 times with WS. Nuclear staining was performed with Hoechst 33342 (Thermo Fisher Scientific, #H3570) diluted 1:5000 v/v in WS. The specimens were washed 3 times with PBS and maintained at 4 °C in PBS supplemented with 0.02% w/v sodium azide.

### Image acquisition and analysis

All microscopy images were acquired with a Leica DMI-6000B fluorescent microscope. In the case of the screening procedure and differentiation experiments, 20X images were acquired with the Matrix Screener mode using a 3 × 3 (screening) or 5 × 5 (other experiments) matrix-layout after auto-focusing.

Efficiency of myogenic differentiation was expressed as the percentage of myogenin positive nuclei over the total number of nuclei. Efficiency of osteogenesis (Alkaline phosphatase positive cells) was estimated as the fraction of alkaline phosphatase area over the total analyzed area.

For the γ-H2AX puncta and RUNX2 intensity assays, 40X images were manually acquired for a total of 5 images per sample. Unbiased image analysis was performed using the Cell Profiler software^65^ using dedicated pipelines.

### SDS-PAGE, western blot and analysis

Cells were harvested without serum-starvation and lysed by scraping in lysis buffer (150 mM NaCl, 50 mM Hepes, 1% v/v Triton X-100, 1 mM EDTA, 1% v/v NP-40) supplemented with 1 mM sodium orthovanadate, 1 mM NaF, 1 mM phenylmethylsulfonyl fluoride, 1X Protease Inhibitor Cocktail (Sigma-Aldrich, #P8340), 1X Phosphatase Inhibitor Cocktail 2 (Sigma-Aldrich, #P5726), 1X Phosphatase Inhibitor Cocktail 3 (Sigma-Aldrich, #P0044) and incubated in ice for 30 minutes. To allow the total swelling of the nuclear compartment the lysates were sonicated using a probe sonicator and then centrifuged at 15,500 x g at 4 °C for 30 minutes. Protein quantitation was assessed with Bradford Bio-Rad Protein Assay Dye Reagent Concentrate (Bio-Rad, #5000006).

Supernatant was treated with 1X NuPAGE™ LDS Sample Buffer (4 × ) (Thermo Fisher Scientific, #NP0007) for 5 minutes at 95 °C. 15 μg of proteins were loaded into the slots of SDS-polyacrylamide gel (Bio-Rad, #5671085, #4561096) and subjected to electrophoresis. Protein blotting was performed on Trans-Blot® Turbo™ Mini/Midi Nitrocellulose Transfer Packs (Bio-Rad, #1704158, #1704159) trough Trans-Blot® Turbo™ Transfer System (Bio-Rad). For ATM kinase western blot 60 μg of proteins were loaded on a manually casted 6% gel and blotted over night at 25 V in immersion transfer system loaded with 20% v/v methanol tris-glycine transfer buffer. The correct protein transfer was evaluated through 1 mg/ml in 5% v/v acetic acid Ponceau S solution (Sigma Aldrich, #P3504). Membranes were washed with TBS-0.1% v/v Tween 20 (TBS-T) until completely clear and blocked with 5% w/v non-fat dry milk in TBS-T.

Primary antibodies were incubated over night at 4 °C in appropriate buffer and dilutions: rabbit anti-RUNX2 (D1L7F) (Cell Signaling Technology, #12556, 1:1000 v/v in 5% w/v BSA/TBS-T), mouse anti-vinculin [SPM227] (Abcam, ab18058, 1:2000 v/v in 5% w/v BSA/TBS-T), rabbit anti-phospho-Smad1 (Ser463/465)/Smad5 (Ser463/465)/Smad9 (Ser465/467) (D5B10) (Cell Signaling Technology, #13820, 1:1000 v/v in 5% w/v BSA/TBS-T), rabbit anti-Smad1 (D59D7) (Cell Signaling Technology, #6944, 1:1000 v/v in 5% w/v milk/TBS-T), rabbit anti-phospho-p38 MAPK (Thr180/Tyr182) (Cell Signaling Technology, #9211, 1:1000 v/v in 5% w/v BSA/TBS-T), rabbit anti-p38 MAPK (Cell Signaling Technology, #9212, 1:1000 v/v in 5% w/v milk/TBS-T), mouse anti-ATM pS1981 (Rockland, #200-301-500, 1:1000 v/v in 5% w/v BSA/TBS-T), mouse anti-ATM [2C1 (1A1)] (Abcam, ab78, 1:1000 v/v in 5% w/v milk/TBS-T), mouse anti-p21 (F-5) (Santa Cruz, sc6246, 1:1000 v/v in 5% w/v milk/TBS-T), rabbit anti- p44/42 MAPK (Erk1/2) (137F5) (Cell Signaling Technology, #4695, 1:4000 v/v in 5% w/v milk/TBS-T), rabbit anti- Phospho-p44/42 MAPK (Erk1/2) (Thr202/Tyr204) (Cell Signaling Technology, #9101, 1:4000 v/v in 5% w/v BSA/TBS-T).

Membranes were washed 3 times with TBS-T for 5 minutes and incubated for 1 hour at RT with secondary antibody: Goat Anti-Mouse IgG (H + L)-HRP Conjugate (Bio-Rad, #1706516, 1:3000 v/v in 3% w/v milk/TBS-T), Goat Anti-Rabbit IgG (H + L)-HRP Conjugate (Bio-Rad, #1706515, 1:3000 v/v in 3% w/v milk/TBS-T). Membranes were washed 3 times with TBS-T for 5 minutes.

HRP chemiluminescence was acquired with a FUJIFILM LAS-3000 Imager system using Clarity™ Western ECL Substrate (Bio-Rad, #170–5061).

Densitometric quantitation of bands was performed with ImageJ (NIH) software. Image brightness and contrast were regulated with the *Auto* function in ImageJ. Cropped image are reported in Figures. The complete gels are available in Supplementary Figures.

### RNA extraction, retro-transcription, real time PCR and analysis

RNA extraction was performed using the NucleoZOL reagent (Macherey-Nagel, #740404) or QIAGEN RNeasy Mini Kit (QIAGEN, #74106) according to manufacturer instructions. The RNA pellet was resuspended in 40 μl of dd-H_2_O. The concentration was evaluated using NanoDrop™ Lite Spectrophotometer (Thermo Fisher Scientific) and the samples were further diluted with ddH_2_O to 100 ng/μl.

Retro-transcription to cDNA was performed on 1 μg of total RNA using the PrimeScript™ RT Reagent Kit (Perfect Real Time) (Takara, #RR037A) according to manufacturer instructions. The reaction was performed using the GeneAmp PCR System 9700 (Applied Biosystems).

Real Time PCR was performed on 100 ng of cDNA (2 μl per reaction) using the TB Green™ Premix Ex Taq™ (Tli RNase H Plus) (Takara, #RR420A) according to manufacturer instructions. The reaction was performed using the StepOne Real-Time PCR System (Thermo Fisher Scientific) or CFX96 System (Bio-Rad).

The primers used in the work are the following: Runx2 FWD 5′-GAAATGCCTCCGCTGTTATG-3′; Runx2 REV 5′-AGGTGAAACTCTTGCCTCGTC-3′; Sp7 FWD 5′-GTCCTCTCTGCTTGAGGAAGAA-3′; Sp7 REV 5′-TCTTTGTGCCTCCTTTCCCC-3′; Bglap FWD 5′-GCAATAAGGTAGTGAACAGACTCC-3′; Bglap REV 5′-GTTTGTAGGCGGTCTTCAAGC-3′; Alpl FWD 5′-ATCTTTGGTCTGGCTCCCATG-3′; Alpl REV 5′-TTTCCCGTTCACCGTCCAC-3′; Myod1 FWD 5′-ACTGCTCTGATGGCATGATGG-3′; Myod1 REV 5′-TAGTAGGCGGTGTCGTAGCC-3′; Myog FWD 5′-GTGAATGCAACTCCCACAGC-3′; Myog REV 5′-CGCGAGCAAATGATCTCCTG-3′; Sox9 FWD 5′-GGAAGTCGGTGAAGAACGGA-3′; Sox9 REV 5′-AGATTGCCCAGAGTGCTCG-3′.

Relative expression was evaluated using the 2^−ΔΔCt^ method^66^.

### Cytofluorimetry analysis of DNA content

Cells were harvested with Lonza™ Trypsin-Versene™-Trypsin-EDTA (Fisher Scientific, #BE17-161E) and centrifuged at 300 x g for 5 minutes at 4 °C. Cells were washed with PBS and centrifuged again.

Samples were resuspended in 250 μl of PBS, fixed and permeabilized adding 250 μl of ice-cold methanol-acetone (4:1 v/v) in gentle vortexing and incubated for 30 minutes in ice. Cells were centrifuged at 1,200 x g for 10 minutes at 4 °C and washed with PBS.

Cells were resuspended in 500 μl of PBS with 100 μg/ml Ribonuclease A (Sigma Aldrich, #R6513) and incubated for 20 minutes at RT. DNA staining was performed adding 25 μg/ml of propidium iodide (Sigma Aldrich, #P4170), incubated for 20 minutes at RT. DNA content was assessed using a FACSCalibur cytofluorimeter (BD Biosciences). 10,000 events per sample were acquired. Data analysis was performed with FlowJo X Software.

### Biological reproducibility and statistical analysis

All the experiments have been conducted on at least 3 independent replicates derived from 3 cell line batches (n = 3). In addition, for immunofluorescence and ALP staining experiments, for each biological replicate we performed three technical replicates to assess the reproducibility of the assay. Real time analyses have been performed in two technical replicates loading the cDNA in two different PCR wells. Data are presented as mean ± standard error of the mean (SEM). Statistical significance was assessed using Student’s t-test, One-Way ANOVA or Two-Way ANOVA according to the data set. Differences were considered statistically significant when p-value < 0.05

## Supplementary information

Osteogenic differentiation of skeletal muscle progenitor cells is activated by the DNA damage response

## Data Availability

All data produced during this work are available from corresponding authors on reasonable request.
