# Supplementary material for: Osteogenic differentiation of skeletal muscle progenitor cells is activated by the DNA damage response
Source: Sci Rep. 2019 Apr 1;9:5447. doi: 10.1038/s41598-019-41926-3 (PMC6443689; doi:10.1038/s41598-019-41926-3)
Supplement: Supplementary file 1 — Osteogenic differentiation of skeletal muscle progenitor cells is activated by the DNA damage response [file 41598_2019_41926_MOESM1_ESM.pdf]

Osteogenic differentiation of skeletal muscle progenitor cells is activated by the DNA damage response

Authors

M. Rosina [1 #], F. Langone [1 #], G. Giuliani [1], A. Cerquone Perpetuini [1], A. Reggio [1], A. Calderone [1], C. Fuoco [1], L. Castagnoli [1], C. Gargioli [1 *], G. Cesareni [1,2 *]

Affiliation

[1] Department of Biology, University of Rome "Tor Vergata", Rome, Italy

[2] Fondazione Santa Lucia Istituto di Ricovero e Cura a Carattere Scientifico (IRCCS), Rome, Italy

# Equal contribution.

* Corresponding authors: G.C. (cesareni@uniroma2.it), C.G. (cesare.gargioli@uniroma2.it)

Supplementary figures

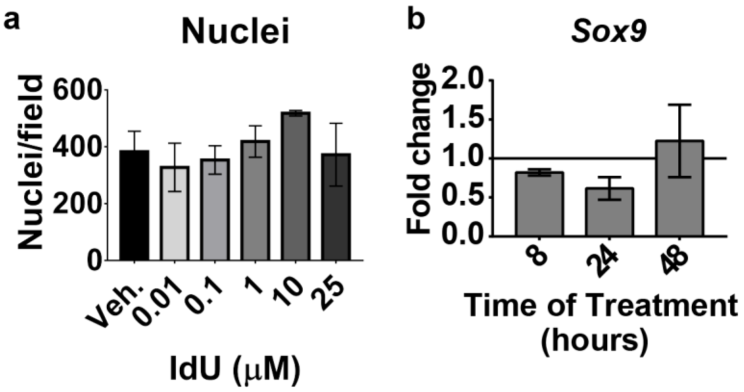

Supplementary Fig. S1: Quantitation of nuclei in differentiation assay and RT-qPCR analysis of Sox9. a) Quantitation of nuclei stained with DAPI in the differentiation assay in Fig. 1 a. Data are presented as mean of nuclei per field ± SEM. Statistical significance was assessed by a One-Way Anova test. n=3. **b)** Real time PCR analysis of *Sox9* in MABs treated with vehicle or 25 μM IdU. Fold change calculation was performed using the $2^{-\Delta\Delta Ct}$ method relative to vehicle treated sample (horizontal line = 1). Data are presented as means ± SEM. Statistical analysis was performed by a Two-Way Anova test. n=3.

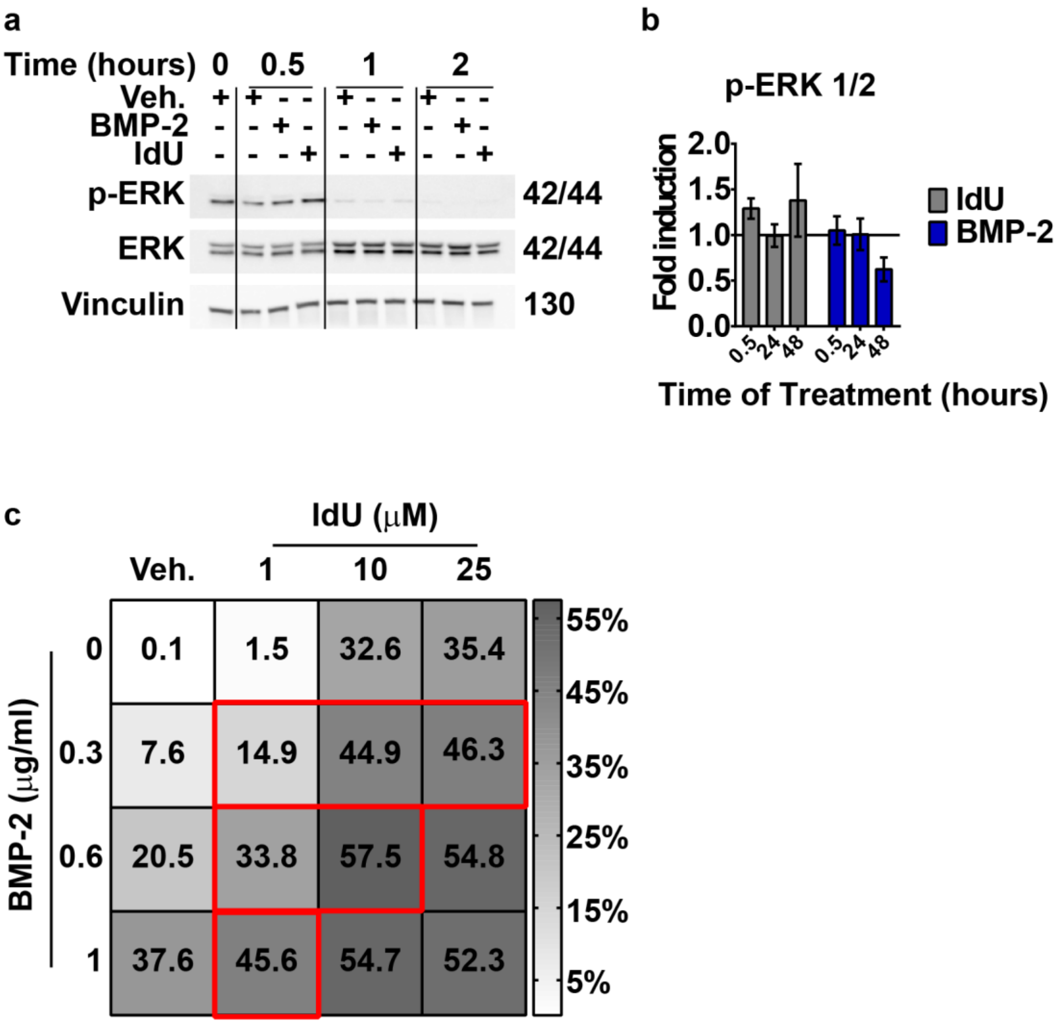

Supplementary Fig. S2: Western blot of p-ERK1/2 and BMP-2/IdU synergy. a) Representative western blot of p-ERK1/2 MAPK. The complete gels are available at Figure S8 **b)** Densitometric quantitation of bands in panel **a**. Data are represented as fold induction relative to vehicle-treated sample (horizontal line = 1). Statistical significance was assessed by a Two-Way Anova test. n=3. **c)** ALP positive area quantitation of the synergy experiment in Figure 3 i. ALP positive area was quantified with the Cell Profiler software. Data are presented as mean percentage of ALP positive

area over the total field area. Red-contoured samples showed an additive effect between IdU and BMP-2.

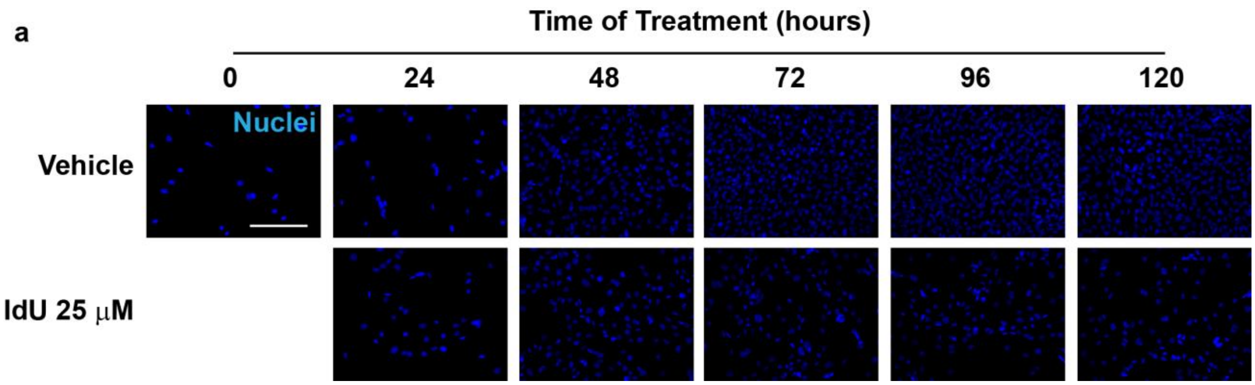

Supplementary Fig. S3: MABs proliferation analysis upon IdU treatment at low seeding density. a) Representative images of MABs growth curve. Nuclei were stained with Hoechst 33342. Scale bar 200 μm.

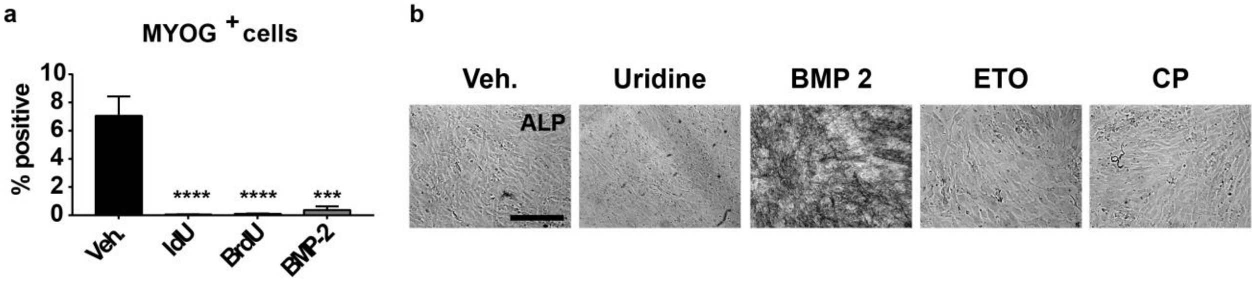

Supplementary Fig. S4: Myogenic differentiation of IdU, BrdU and BMP-2 treated MABs. a) Myogenin positive cells quantitation of images in Fig. 5 a. Data are presented as percentage of MYOG positive cells over the total cells in the image filed ± SEM. Statistical significance was assessed by a One-Way ANOVA test. *** $p<0.001$; **** $p< 0.0001$; n=3. **b)** ALP staining of MABs treated with vehicle (Veh.), 25 μM uridine, 1 μg/ml BMP-2, 0.5 μM etoposide and cisplatin.

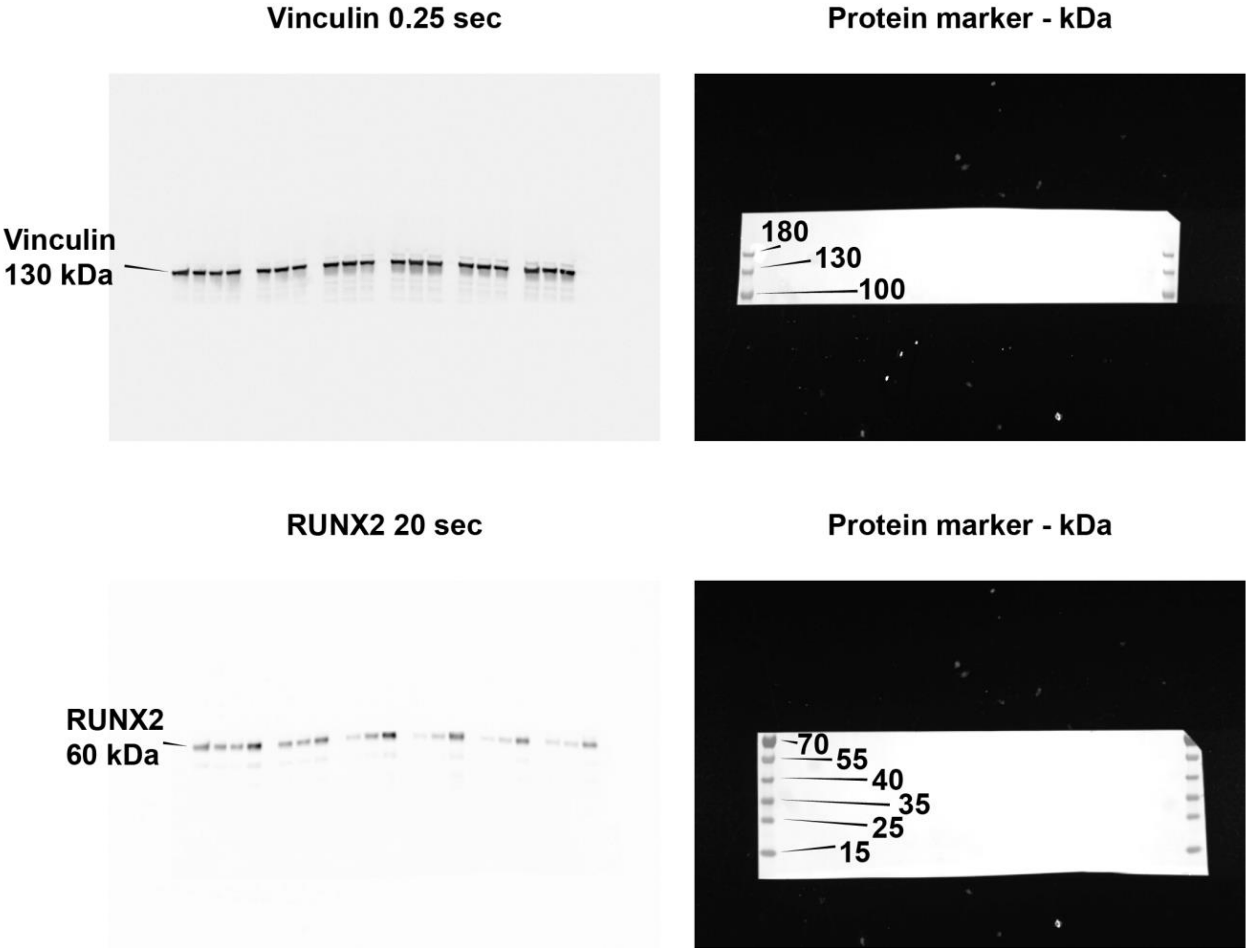

Supplementary Fig. S5: complete western blot of bands in Fig. 2 a

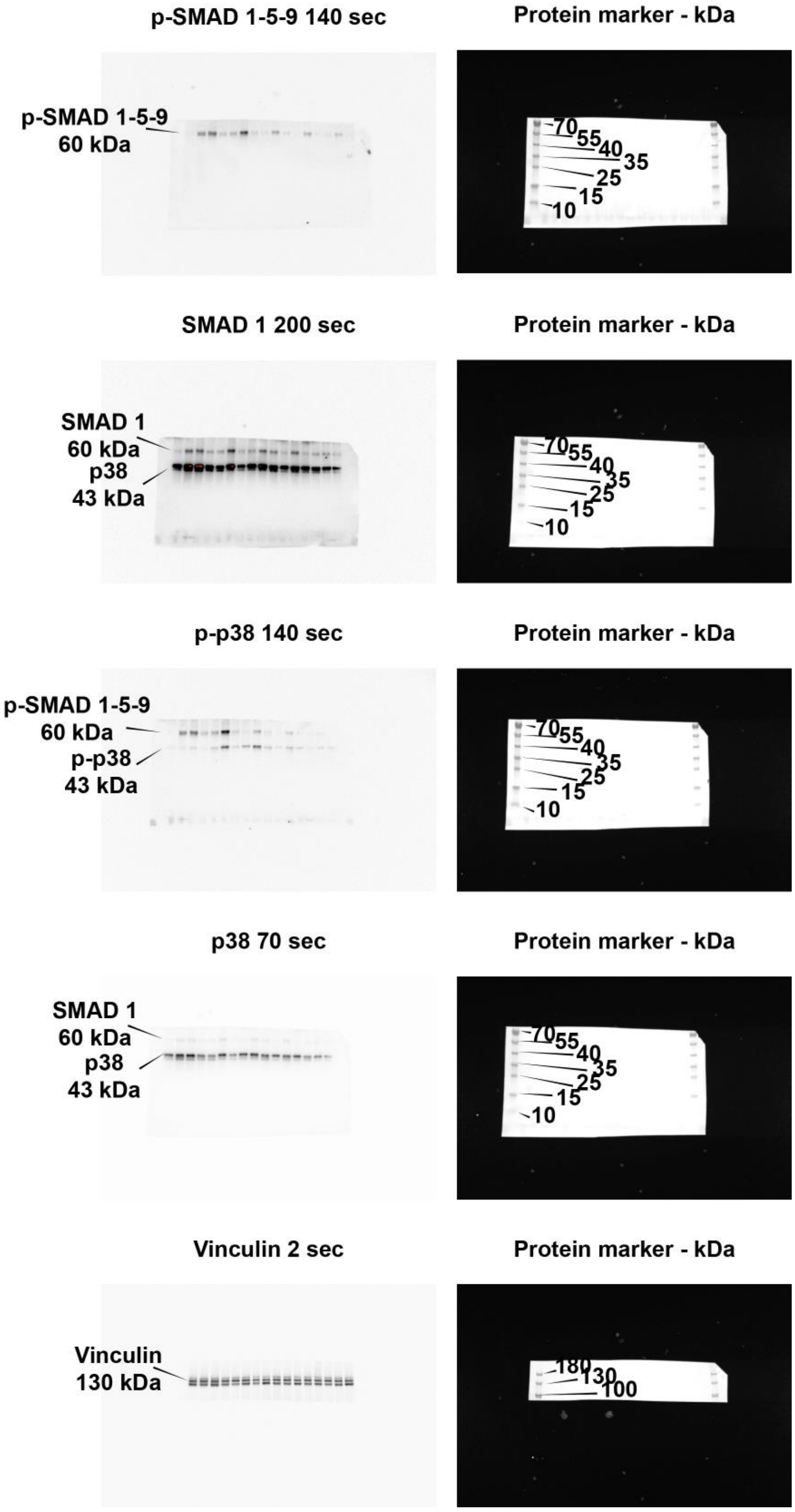

Supplementary Fig. S6: complete western blot of bands in Fig. 3 b

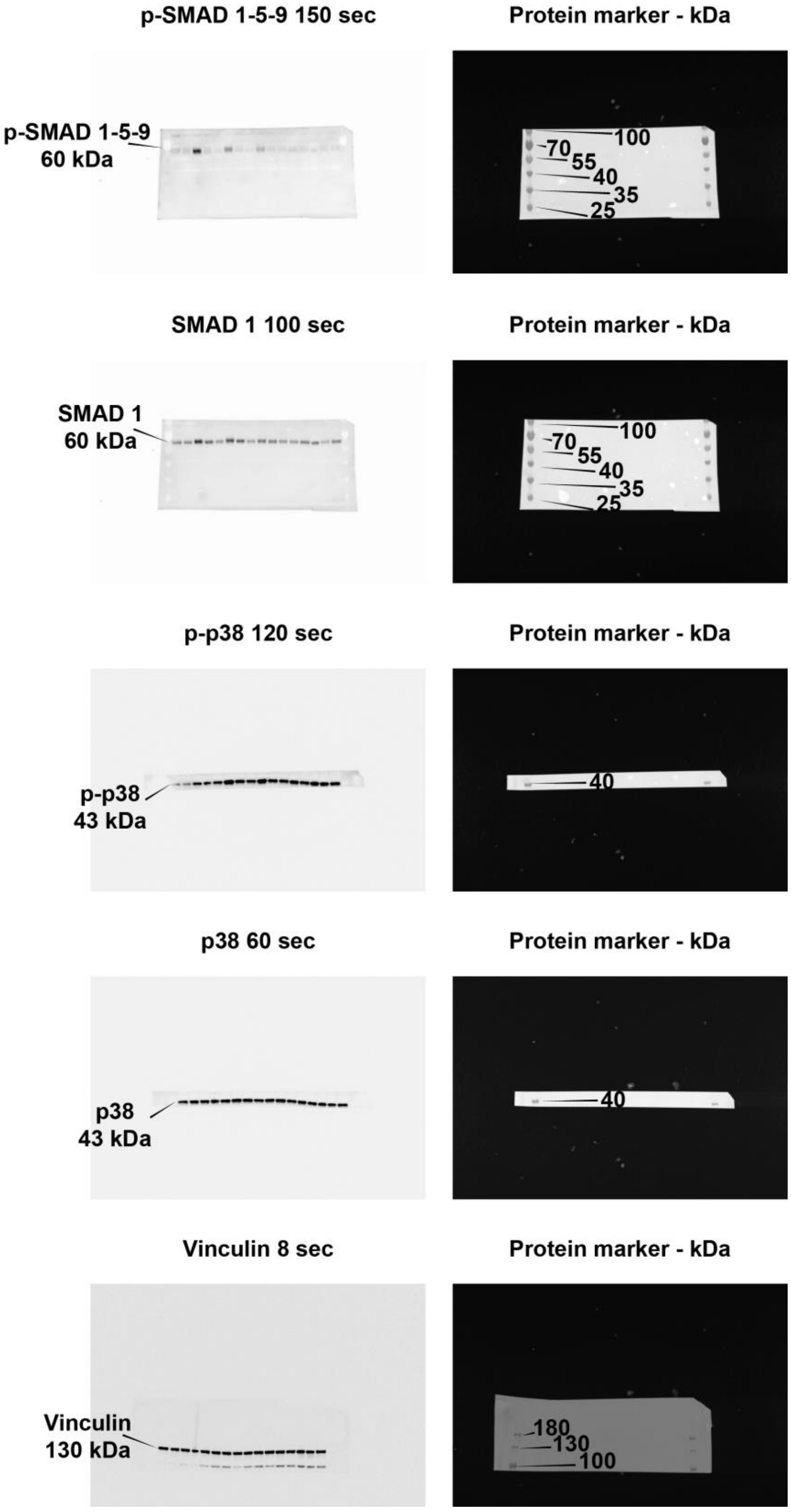

Supplementary Fig. S7: complete western blot of bands in Fig. 3 c

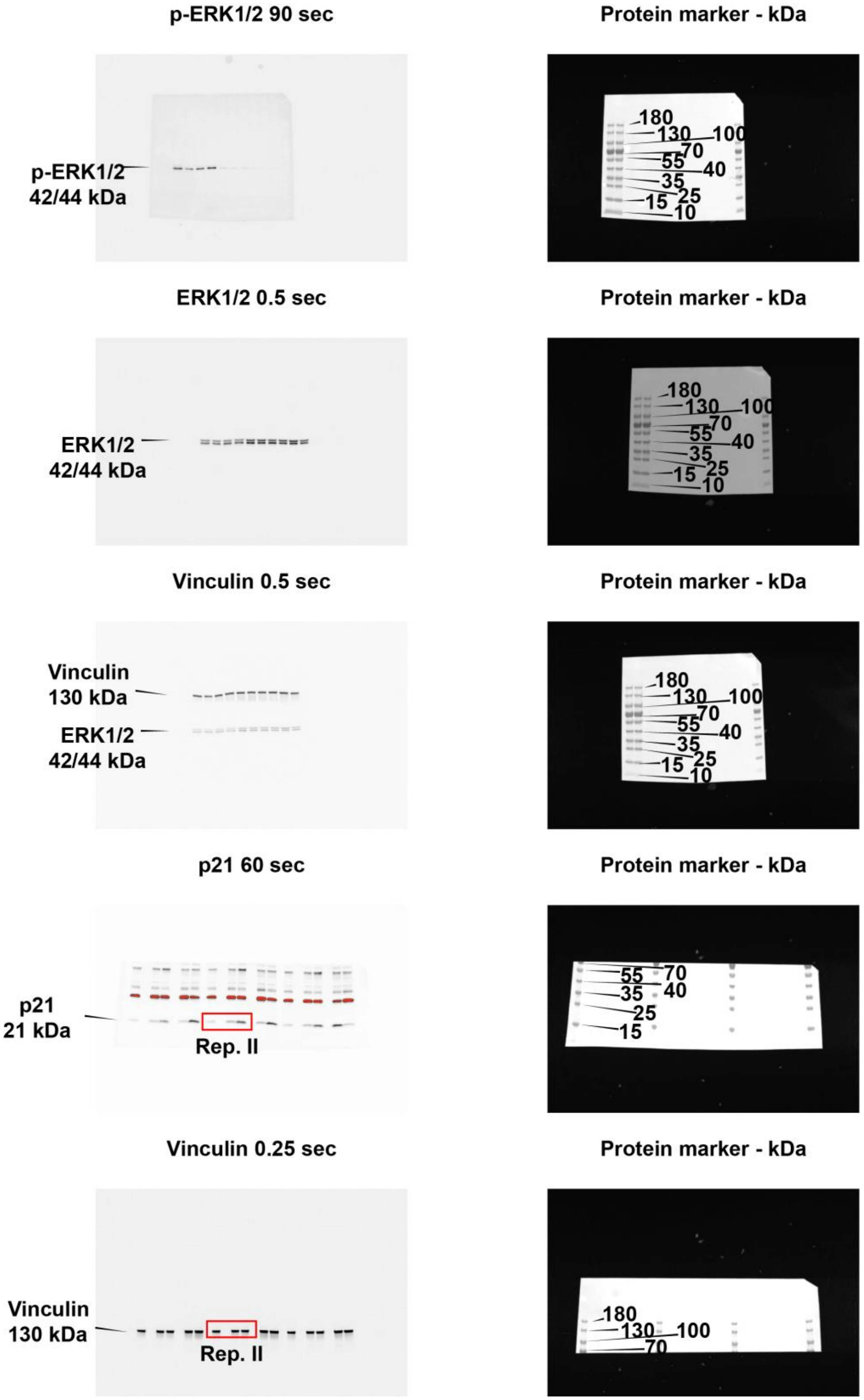

Supplementary Fig. S8: complete western blot of bands in Fig. S2 a-b and Fig. 4 e

p-ATM 210 sec

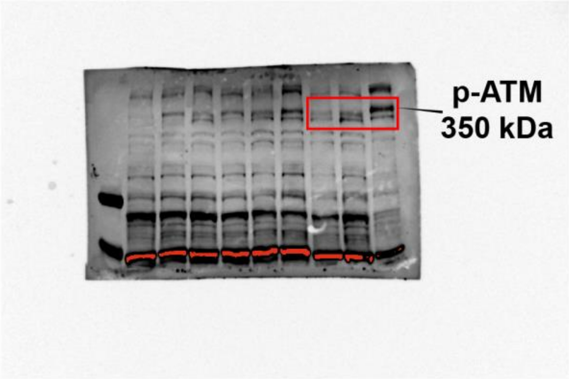

Protein marker - kDa

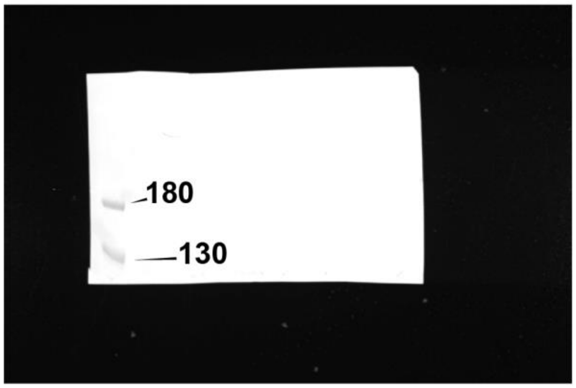

ATM 30 sec

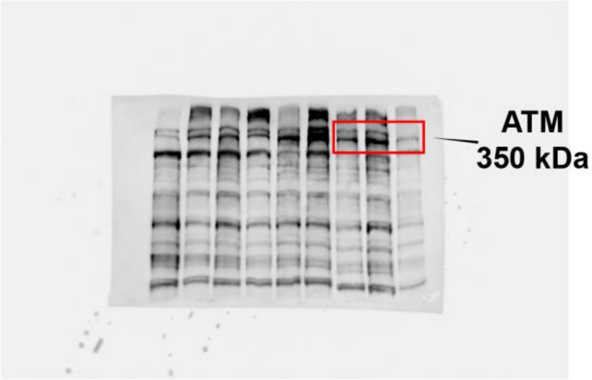

Protein marker - kDa

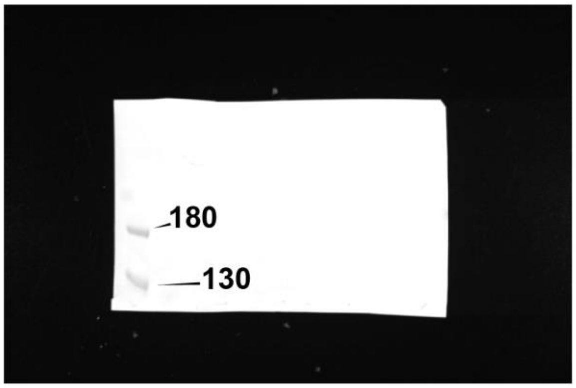

Vinculin 0.5 sec

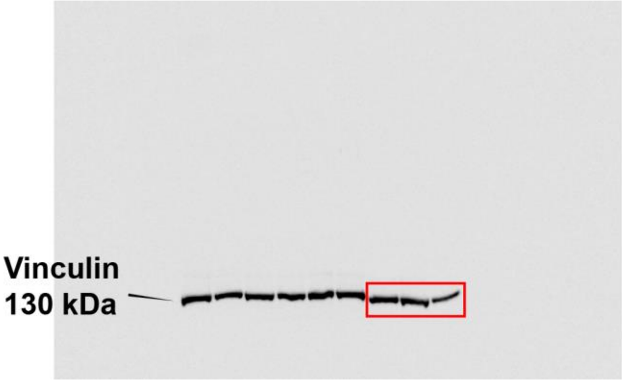

Protein marker - kDa

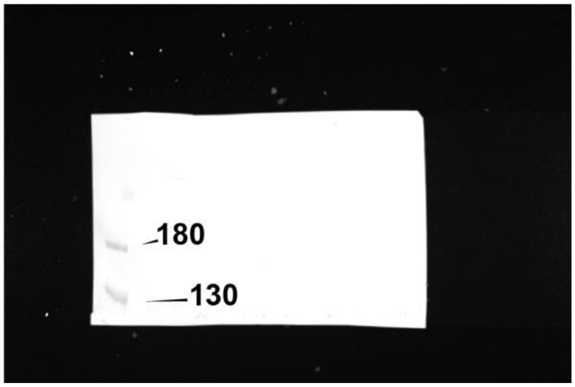

Supplementary Fig. S9: complete western blot of bands in Fig. 5 c

Supplementary tables

Supplementary Table ST1. List of inducers of MABs differentiation into osteoblasts

Chemical name	Therapeutic group	Mechanism of action
Idoxuridine	Antiviral	Nucleic acid synthesis inhibitor
Isotretinoin	Cystic acne	unknown

Supplementary Table ST2. List of inducers of MABs differentiation in skeletal muscle

Chemical name	Therapeutic group	Mechanism of action
Doxylamine succinate	Antihistaminic	H1 antagonist

Supplementary Table ST3. List of inhibitors of MABs differentiation in skeletal muscle

Chemical name	Therapeutic group	Mechanism of action
Sulfathiazole	Antibacterial	Inhibitor of folic acid synthesis
Levodopa	Antiparkinsonian	Tyrosine aminotransferase inhibitor
Troleandomycin	Antibacterial	Ribosomal protein synthesis inhibitor
Acetazolamide	Diuretic	Carbonic anhydrase inhibitor
Clemizole hydrochloride	Antihistaminic	H1 antagonist
Dilazep dihydrochloride	Vasodilatator	Adenosine uptake inhibitor
Androsterone	Antihypertensor	-

Supplementary Table ST4. Two-Way ANOVA statistics of the synergism experiment in Fig. 3 i. The Two-Way ANOVA test has been performed with GraphPad Prism 6 software. The “Interaction” term (highlighted in yellow) refers to the co-treatment effect between BMP-2 and IdU. p=0.0013 supports the synergism hypothesis between the two groups.

**Table Analyzed**	**BMP-IdU Synergy**				
Two-way ANOVA	Ordinary				
Alpha	0,05				
**Source of Variation**	**% of total variation**	**P value**	**P value summary**	**Significant?**	
Interaction	5,826	0,0013	**	Yes	
BMP-2	34,44	<0,0001	****	Yes	
IdU	48	<0,0001	****	Yes	
**ANOVA table**	**SS**	**DF**	**MS**	**F (DFn, DFd)**	**P value**
Interaction	1854	9	206	F (9, 64) = 3,531	P=0,0013
BMP-2	10961	3	3654	F (3, 64) = 62,63	P<0,0001
IdU	15275	3	5092	F (3, 64) = 87,29	P<0,0001
Residual	3733	64	58,33		
Number of missing values	0				
